# Complete genome sequence of *Stutzerimonas balearica* JWOYS.421, a denitrifying strain isolated from the Hong Kong oyster (*Magallana hongkongensis*)

**DOI:** 10.1128/mra.00404-26

**Published:** 2026-05-18

**Authors:** J. H. Y. Wong, K. M. Leung, G. K. K. Lai, B. D. Russell, S. D. J. Griffin

**Affiliations:** 1 Shuyuan Molecular Biology Laboratory, The Independent Schools Foundation Academy, Hong Kong SAR, China; 2 The Swire Institute of Marine Science and Area of Ecology and Biodiversity, School of Biological Sciences, The University of Hong Kong, Hong Kong SAR, China; 3 Institute for Climate and Carbon Neutrality, The University of Hong Kong, Hong Kong SAR, China; Montana State University, Bozeman, Montana, USA

**Keywords:** *Stutzerimonas balearica*, denitrification, Dpd operon, *Magallana hongkongensis*

## Abstract

*Stutzerimonas balearica* strain JWOYS.421 was recovered from the shell of a Hong Kong oyster (*Magallana hongkongensis*) collected from coastal waters in Hong Kong. Its complete genome, a single circular chromosome of 4,405,581 bp (64.80% G+C), was established through hybrid assembly.

## ANNOUNCEMENT

Genus *Stutzerimonas* was proposed in 2022 for species formerly grouped within the *Pseudomonas stutzeri* complex, whose denitrifying activity had been observed as early as 1895 (1, 2). *Stutzerimonas balearica* is a Gram-negative, mesophilic, rod-shaped facultative anaerobe previously isolated from oil-polluted coastal sediment in the Balearic Islands (3). Exhibiting halotolerance up to 8.5% NaCl, its metabolic versatility has revealed it a promising candidate for marine bioremediation (3–5).

Here, *Stutzerimonas balearica* JWOYS.421 was isolated from the shell of a Hong Kong oyster (*Magallana hongkongensis*) from Lau Fau Shan (22.4682°N, 113.9845°E). One gram of crushed shell was vortexed with 19 mL saline (0.9% w/v) and allowed to settle for ~5 min. A 50 µL aliquot of the clearer supernatant was then added to 2 mL of A+ medium (6), and the mixture incubated overnight with shaking. After a 10,000-fold dilution with saline, 100 µL of the culture was spread onto denitrification medium (DM) agar containing 3.5% w:v NaCl (7) for overnight incubation. Resultant colonies were confirmed to reduce nitrate and nitrite (8) and selected isolates passaged to purity on DM agar. A single colony of JWOYS.421 was streaked onto DM agar before harvesting overnight growth for DNA extraction using Qiagen’s DNeasy PowerSoil Pro kit. All agar/broth incubations were at 27°C.

Paired-end short-read libraries (NEBNext Ultra DNA Library Prep Kit, Illumina, Inc.) were sequenced via the NovaSeq 6000 platform using an SP PE250 flowcell and v1.5 Reagent Kit. A total of 9,169,642 raw single reads (mean length 250 bp) (SRX30846274) were quality-filtered and trimmed using TrimGalore! v0.6.7 (stringency:3; -e:0.2), producing 4,573,296 read pairs (mean length 247 bp, total ~1,129.6 Mbp). Long-read libraries, prepared from the same extracted DNA using the Rapid Barcoding Kit SQK-RBK004 without size selection, were sequenced via Oxford Nanopore’s Spot-ON Flow Cell (vR9.4.1), MinION sequencer, and MinKNOW v20.06.2 software, with base-calling by Guppy v6.1.55 (high-accuracy mode). The final long-read data set of 288,403 raw reads (mean length 3,694 bp) (SRX30846275) was trimmed by Filtlong v0.2.1 (https://github.com/rrwick/Filtlong) (min_length: 2,000; target_bases: 500Mbp; length_weight: 10), selecting 71,777 reads (mean length 9,394 bp, N50 9,910; total ~674.3 Mbp) to scaffold the assembly. Default parameters were used for all software, unless otherwise specified.

Illumina and MinION data sets were combined using Trycycler v.0.5.5 (9), with long-read polishing by Medaka v2.1.0 and short-read polishing by Polypolish v0.6.0 (short-read coverage 256×) (10, 11), yielding a single circular chromosome (4,405,581 bp, 64.80% G+C) rotated to begin *dnaA* (A0A8D3XX96) with mean long-read coverage 153× (judged 99.34% complete with 0.96% contamination by CheckM v1.2.4 [12]), which was submitted to NCBI PGAP v6.10 (13) for annotation and to JSpecies v5.0.2 (14) for taxonomic assignment. *Stutzerimonas balearica* JWOYS.421 shares an average nucleotide identity (ANIu by EZBioCloud [15]) of 98.30% with *Stutzerimonas balearica* DSM 6083 (CP007511.1). Genes linked to denitrification, predicted via PGAP v6.10 and by BLASTp with *Stutzerimonas stutzeri* A1501 (CP000304.1) (16), are shown in Table 1. Nitrite reductase NirS, encoded at ACRYHF_17195, belongs to clade 1a (17, 18), while NirK is not present. A Dpd operon enabling DNA modification with 7-deazaguanine derivatives (19), which is absent in DSM 6083 and A1501, is found at loci ACRYHF_11555 to ACRYHF_11630.

**TABLE 1:** Predicted genes^*a*^ in *Stutzerimonas balearica* JWOYS.421 linked to denitrification

Gene(s)	Locus (NCBI)	Activity
*nasD nasE*	ACRYHF_00990, ACRYHF_00900	Soluble NADH-dependent nitrite reductase
*ntrC ntrB*	ACRYHF_01850, ACRYHF_01855	Nitrogen regulation protein NR(I), NR(II)
*narKMXL narGHJI*	ACRYHF_03580 to ACRYHF_03565 ACRYHF_03585 to ACRYHF_03600	Nitrate transport and regulation, dissimilatory nitrate reductase
*putative dnrE*	ACRYHF_04205	Transcription factor regulating respiratory nitrate reduction
*norR norCB norD*	ACRYHF_04175 ACRYHF_04265 to ACRYHF_04275	Nitric oxide reductase
*napEDABC*	ACRYHF_06300 to ACRYHF_06280	Periplasmic nitrate reductase
*norCBQD*	ACRYHF_08815 to ACRYHF_08830	bc-type nitric oxide reductase
*nirBD*	ACRYHF_09940, ACRYHF_09945	Siroheme-containing nitrite reductase
*nasTS*	ACRYHF_12175, ACRYHF_12170	Control of assimilatory nitrate/nitrite reduction
*nasAB*	ACRYHF_12180, ACRYHF_12195	Assimilatory nitrate/nitrite reductase
*nirMCFDLGH*	ACRYHF_17190 to ACRYHF_17160	Biosynthesis and maturation of the cytochrome cd1 nitrite reductase NirS
*nirS*	ACRYHF_17195	Nitrite reductase NirS
*nirQOP*	ACRYHF_17200 to ACRYHF_17210	Reductase regulation
*nirJENY*	ACRYHF_17215 to ACRYHF_17230	Biosynthesis of the heme d1 cofactor
*nosRZDFYL-tatA*	ACRYHF_17290 to ACRYHF_17260	Nitrous oxide reductase

^*a*^ By NCBI PGAP v6.10 (11) and by BLASTp with *Stutzerimonas stutzeri *A1501 (CP000304.1) (13).

## Data Availability

Sequencing data for *Stutzerimonas balearica* JWOYS.421 is available through NCBI under GenBank assembly GCA_051468625.1 and GenBank accession CP195794. Raw reads may be found under SRX30846274 (NovaSeq) and SRX30846275 (MinION). BLASTp comparison data between *Stutzerimonas stutzeri* A1501 and *Stutzerimonas balearica* JWOYS.421 can be found at https://doi.org/10.6084/m9.figshare.31901167.
